# Comparison of the Safety and Efficacy of Interferon Alpha-2a and Cyclosporine-A When Combined With Glucocorticoid in the Treatment of Refractory Behçet’s Uveitis: A Randomized Controlled Prospective Study

**DOI:** 10.3389/fphar.2021.699903

**Published:** 2021-07-19

**Authors:** Yujing Qian, Yi Qu, Fei Gao, Minghang Pei, Anyi Liang, Junyan Xiao, Chan Zhao, Meifen Zhang

**Affiliations:** ^1^ Department of Ophthalmology, Peking Union Medical College Hospital, Chinese Academy of Medical Sciences and Peking Union Medical College, Beijing, China; ^2^ Department of Ophthalmology, The First Affiliated Hospital of Zhengzhou University, Zhengzhou, China; ^3^ Key Laboratory of Ocular Fundus Diseases, Chinese Academy of Medical Sciences and Peking Union Medical College, Beijing, China

**Keywords:** Behçet’s disease, interferon alpha-2a, cyclosporine-A, uveitis, randomized controlled trial

## Abstract

**Purpose:** To evaluate and compare the efficacy and safety of interferon alpha-2a (IFN-α2a) and cyclosporine-A (CsA) in patients with refractory Behçet’s uveitis (BU).

**Methods:** In this 12-month randomized, controlled, prospective trial, 26 participants (44 eyes) completed the study. Patients were randomly allocated to the IFN-α2a or CsA groups. All patients in both groups received a standardized prednisone burst and tapering schedule as per protocol. The primary outcome measures were response rate, complete remission rate, and tolerance rate. The secondary outcome measures included time to achieve complete remission, the logarithm of the minimum angle of resolution (logMAR) of best-corrected visual acuity (BCVA), and Behçet’s disease ocular attack score 24 (BOS24). T-tests and non-parametric tests were used to compare quantitative variables, and chi-square tests were performed to compare qualitative variables.

**Results:** The response and complete remission rates were 85.7% (12/14 patients) and 50.0% (7/14 patients) in the IFN-α2a group, compared with 66.7% (8/12 patients) and 25.0% (3/12 patients) in the CsA group, respectively (*p* > 0.05). Complete remission was achieved at 3.3 and 7.0 months after initiation of IFN-α2a and CsA (*p* = 0.023). LogMAR BCVA significantly improved 1 month after IFN-α2a initiation (23 eyes) (*p* = 0.002), and this beneficial effect remained statistically significant during the entire follow-up period (*p* < 0.05); however, this improvement was not observed in the CsA group (21 eyes). At the endpoint, LogMAR BCVA in the IFN-α2a group was significantly better (0.22 vs. 0.31, *p* = 0.031) with a higher improvement rate (60.9 vs. 47.6%, *p* > 0.05). Moreover, compared to the CsA group, more eyes in the IFN-α2a group had a lower BOS24 score (87.0 vs. 57.1%, *p* = 0.042). None of the patients had any side effects that influenced the medication adherence.

**Conclusion:** Compared to CsA plus corticosteroid, IFN-α2a plus corticosteroid appears to induce a better treatment response, a significantly greater improvement in visual acuity, and more stable remission of intraocular inflammation in a 12-month study period.

**Clinical Trial Registration:** Interferon α2a Versus cyclosporine for refractory Behçet’s disease uveitis, NCT03209219.

## Introduction

Behçet’s disease (BD) is a multisystemic chronic inflammatory disease of unknown cause characterized by recurrent oral aphthous ulcers, ocular lesions, genital ulcers, gastrointestinal, and central nervous system manifestations (Greco et al., 2018). Uveitis is one of the most common and debilitating organ impairments, affecting 50–70% of BD patients, and may eventually lead to blindness in 25% of patients despite aggressive treatment (Tugal-Tutkun et al., 2004; Greco et al., 2018). Behçet’s uveitis (BU) classically manifests as recurrent non-granulomatous uveitis involving the posterior segment of the eye with or without anterior segment inflammation (Paovic et al., 2013), and visual loss is determined by accumulative damage to the intraocular structure caused by repeated episodes of acute uveitis attacks (Tugal-Tutkun et al., 2004; Takeuchi et al., 2005). Therefore, it is of great clinical importance to suppress the inflammation during an acute attack and to prevent recurrence in the quiescent phase.

Current treatments for BU mainly include glucocorticoids, conventional immunosuppressants such as cyclosporine-A (CsA) and azathioprine (AZA), and biological agents such as interferon-alpha (IFN-α) and anti-tumor necrosis factor-alpha (anti-TNF-α) agents (Schwartzman, 2016). While high-dose glucocorticoids are recommended as the mainstay treatment for acute ocular attacks, they are not suitable for long-term use because of their adverse effects (Hatemi et al., 2018). Conventional immunosuppressive agents are usually helpful as add-on treatments for persistent uveitis (Mesquida et al., 2014). Unfortunately, up to 41.3% of refractory BU patients show inadequate responses to conventional immunosuppressives even at optimal therapeutic doses; therefore, switching to biologics could be considered (Celiker et al., 2018).

IFN-α2a has long been reported to be effective in BU patients with different genetic backgrounds (Gueudry et al., 2008; Sobac et al., 2010; Lee et al., 2018; Yang et al., 2019). IFN-α2a has the advantage of rapid onset of action and long-term remission, and accumulating evidence suggests that IFN-α2a may be superior to conventional agents because it is usually effective for BU patients refractory to immunosuppressives (Deuter et al., 2010; Park et al., 2015; Kavandi et al., 2016; Diwo et al., 2017; Hasanreisoglu et al., 2017; Shi et al., 2019; Eser-Ozturk and Sullu, 2020). However, all the above-mentioned studies are retrospective observational studies and uncontrolled case series, and to the best of our knowledge, there is still a lack of prospective studies that provide solid evidence for the effectiveness of IFN-α2a in refractory BU. Therefore, a randomized controlled prospective study was conducted to compare the efficacy and safety of IFN-α2a and CsA in the treatment of refractory BU.

## Materials and Methods

### Study Design and Patient Population

This 12-month randomized controlled prospective study was conducted at the Department of Ophthalmology at Peking Union Medical College Hospital between June 2017 and August 2020. All recruited patients with refractory BU were randomly assigned (1:1) to the IFN-α2a or CsA groups using a random number table. The study protocol was approved by the Institutional Review Board of Peking Union Medical College Hospital (approval number: JS-1342) and conducted according to the tenets of the Declaration of Helsinki. Informed consent was obtained from all participants. This study was registered in ClinicalTrials.gov (NCT03209219).

The study population was adult (18 ≤ age ≤ 65) refractory BU patients with acute uveitis attack. BD was diagnosed according to the International Criteria for Behçet’s Disease (ICBD) (Davatchi et al., 2014). Uveitis terminology and anatomic classification were described by the Standardization of Uveitis Nomenclature (SUN) (Jabs et al., 2005). Patients were eligible for the study if they had posterior uveitis or panuveitis acute attacks (≥1 + vitreous haze together with the presence of at least one of the following lesions: retinal vasculitis, retinitis, cystoid macular edema, or papillitis) under a medium dose of oral glucocorticoids (prednisone, no less than 15 mg/day or equivalent) and at least one of the following conventional immunosuppressants: CsA (≥100 mg/day), AZA (≥50 mg/day), cyclophosphamide (CTX, ≥100 mg/day), methotrexate (MTX, ≥15 mg/week), mycophenolate mofetil (MMF, ≥1,000 mg/day), thalidomide (THD, ≥2 mg/day), and tacrolimus (TAC, ≥2 mg/day).

Patients with any of the following conditions were excluded: 1) patients who had previously received any biological agent (e.g., IFN-α, anti-TNF-α agents, anti-human IL-6 receptor antibody), had used CsA but did not tolerate, or had any systematic contraindication (e.g., active peptic ulcer, osteoporosis, infection) that prevent using glucocorticoids; 2) patients with malignancy, pregnant, breast-feeding, mental illness, depression, cognitive impairment, poorly controlled hypertension or diabetes mellitus, alcohol abuse or drug abuse, history of acute or chronic inflammatory joint or autoimmune disease, systemic infectious diseases, including hepatitis B virus, hepatitis C virus, HIV, syphilis, or tuberculosis (TB) infection were also excluded; 3) patients with severe extra-ocular involvement; 4) patients who showed a presence of severe pupillary adhesion, cataract and posterior capsular opacification that obscured the fundus observation, and/or had other ocular diseases, and intraocular surgery in the previous 3 months; and 5) patients with significant laboratory abnormalities in complete blood counts (e.g., white blood cell count < 3,500/mm^3^, platelet count < 100,000/mm^3^, Hgb < 8.5 g/dl), urine tests, liver and kidney function (e.g., creatinine > 1.5 mg/dl, alanine transaminase (ALT) or aspartate transaminase (AST) 2× above the normal) were not eligible.

### Treatments

**FIGURE 1:**
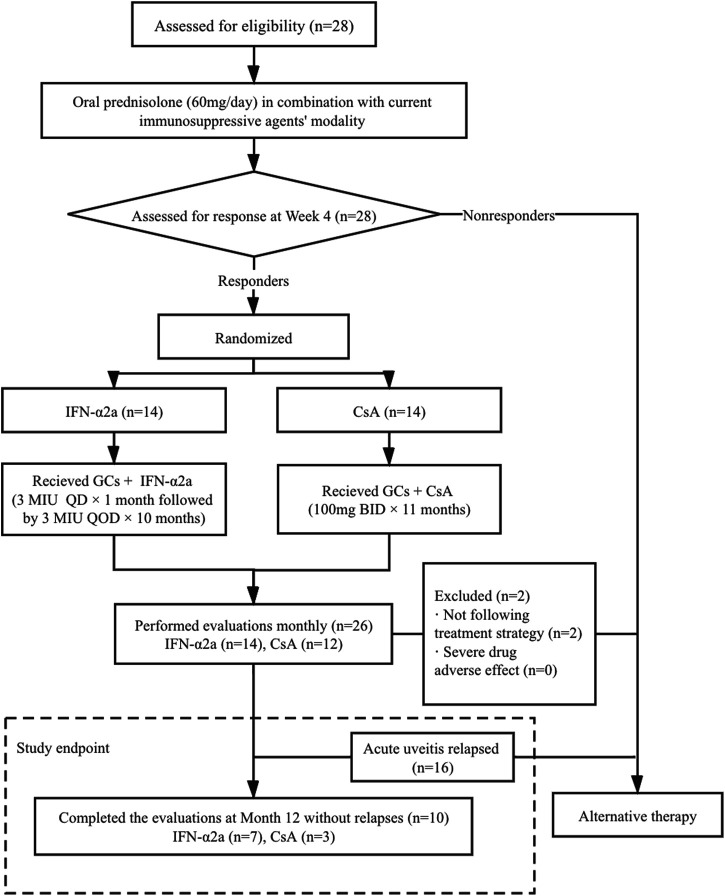
Patient disposition and flow chart of the study. BID, bis in die (twice a day); CsA, cyclosporine-A; GCs, glucocorticoids; IFN-α2a, interferon alpha-2a; MIU, million international unit; QD, quaque die (every day); QOD, quaque omni die (every other day).

As shown in the treatment protocol (Figure 1), oral corticosteroid was up-titrated to 60 mg/day of prednisolone with current immunosuppressant modality, which remained unchanged for the first 4 weeks. Responders who showed an improvement in vitreous haze and chorioretinal inflammation were randomly divided into two groups. In the IFN-α2a group, patients received a daily dose of 3 million international units (MIU) of IFN-α2a (Interfon; 3sbio.inc., Shenyang, China) subcutaneously for 4 weeks, followed by 3 MIU every other day as the maintenance dose. In the CsA group, patients received 100 mg of CsA twice per day during the entire study period. Meanwhile, for all patients in both groups, all other immunomodulating agents were discontinued when IFN-α2a or CsA therapy was initiated, and the dose of prednisolone was tapered from 55 mg/day following the same protocol, that is, reduce 5 mg/day every 10 days to 30 mg/day, reduce 2.5 mg/day every 14 days to 15 mg/day, and it remained unchanged thereafter.

In the case of anterior uveitis, corticosteroid and mydriatic eye drops were allowed to prescribe. Gastric mucosal protective agents, vitamin D, calcium, potassium, and hepatoprotectants were administered when necessary.

### Follow-Up Schedule, Clinical Assessment, and Endpoints

Patients were recommended to visit our center monthly until 12 months after the initiation of 60 mg/day prednisolone, and whenever symptoms suggestive of disease recurrence were noted.

A detailed ophthalmic examination including best-corrected visual acuity (BCVA), intraocular pressure, slit-lamp biomicroscopy, and fundoscopy, were performed at baseline (before the initiation of 60 mg/day prednisolone) and at each follow-up visit. BCVA was examined using standard logarithmic visual acuity charts and then converted to the logarithm of the minimum angle of resolution (LogMAR) for statistical analysis.

An ocular inflammatory attack was defined as a new-onset of intraocular inflammation and/or worsening of preexisting uveitis, necessitating treatment intensification. The severity of ocular inflammation at baseline and each follow-up visit was evaluated using the BOS24 scoring system, which is a novel and more definite tool for scientific analysis (Kaburaki et al., 2014; Tanaka et al., 2016). The BOS24 consists of 24 points describing six parameters of ocular inflammation manifestations, including anterior chamber cells (maximum 4 points), vitreous opacity (maximum 4 points), peripheral fundus lesions (maximum 8 points), posterior pole lesions (maximum 4 points), subfoveal lesions (maximum 2 points), and optic disc lesions (maximum 2 points). Changes in the BOS24 score before and after IFN-α2a or CsA treatment were recorded and compared.

Bone mineral density and infection screening tests were performed at baseline. Blood pressure was measured at baseline and monthly during the study period. Laboratory tests, including complete blood counts, urine tests, and biochemical tests, were performed monthly or bimonthly.

The endpoints of this study were relapse of posterior or pan-uveitis, drug (prednisolone, CsA, or IFN-α2a) withdrawal due to intolerance, and completion of the 12-month follow-up since initiation of 60 mg/day prednisolone.

The primary efficacy outcome measures were the response and complete remission rates. Specifically, treatment response was categorized into complete remission, partial remission, and treatment failure. Complete remission was defined as a decrease in vitreous haze to no more than grade 0.5+ and complete disappearance of signs of active fundus inflammation including retinal infiltrates, hemorrhage, and vascular sheathing (Jabs et al., 2005), without any relapses within the 12-month follow-up. Partial remission was defined as improvement in vitreous haze and chorioretinal inflammation, but it did not reach the standard of complete remission. Treatment failure was defined as vitreous haze or chorioretinal inflammation that remained unchanged or even exacerbated during the study period. The secondary efficacy outcome measures included time to reach complete remission, duration of relapse-free, glucocorticoid-sparing effect, and changes in BCVA and BOS24.

The primary safety outcome measure was the tolerance rate to IFN-α2a or CsA treatment. The secondary safety outcome measures included the incidence of adverse effects, significant abnormal changes in vital signs or laboratory test results, and the adverse effects profile.

### Statistical Analysis

Statistical analysis was conducted using the Macintosh software (version 25.0; IBM Corp. Released 2017. IBM SPSS Statistics for Macintosh, version 25.0. Armonk, NY: IBM Corp.). The Kolmogorov–Smirnov test was used for normality testing. Normal variables are presented as the mean and standard deviation (SD), and non-normal variables as the median and interquartile range (IQR). T-tests were used to compare the means of normally distributed quantitative variables; otherwise, the Mann-Whitney U test was used. The non-parametric Wilcoxon test was used to compare continuous variables. Chi-square tests were used to compare the qualitative data. Statistical significance was set at *p* value of <0.05.

### Sample Size Analysis

Sample size analysis was conducted using PASS 11.0 software (NCSS, LLC). This randomized controlled prospective study was designed to have a statistical power of 80% and a significance level of 5%. Based on our clinical experience and previous studies, we estimated that the primary endpoint of participants, namely, complete remission rate of IFN-α2a and CsA therapy, was 80 and 30% (Kötter et al., 2004), respectively. Given that 10% of subjects may lost to follow-up or drop out, the minimum number was 14 patients for each group.

## Results

### Characteristics of Patients

A total of 28 eligible patients were included in the study from June 2017 (enrollment of the first patient) to August 2020 (the date of the last follow-up visit). Two patients who did not follow the treatment protocol were excluded. Therefore, 26 patients with refractory BU (44 eyes) completed the trial and were included in the analysis. As shown in Table 1, of the 26 included patients, the mean age was 32.2 ± 9.2 years and 24 patients (92.3%) were men, 14 were in the IFN-α2a group, and 12 were in the CsA group. Eye involvement was bilateral in 18 patients (69.2%). Panuveitis was the most common ocular manifestation, presenting in 26 (58.1%) eyes, and posterior uveitis was present in 18 (41.9%) eyes. The median duration of BD was 25.0 months (range, 1–156 months). Recurrent oral ulcers were present in all patients (100.0%), followed by erythema nodosum in 11 patients (44.0%), genital ulcers in 10 patients (38.5%), pseudo-folliculitis in 7 patients (26.9%), arthritis in 2 patients (8.0%), and thrombophlebitis and perianal abscess each in 1 patient (4.0%). After treatment, no new extraocular manifestations were detected in either group.

**TABLE 1:** Baseline features of 26 patients with refractory BU.

	Total (n = 26)	IFN-α2a (n = 14)	CsA (n = 12)
Age (years), $\bar{x}$±s	32.2 ± 9.2	32.1 ± 7.8	32.2 ± 10.9
Male, n (%)	24 (92.3)	13 (92.9)	11 (91.7)
Duration of BD (months), M (IQR)	25.0 (20.0–36.0)	24.5 (19.5–30.0)	32.5 (21.0–49.0)
Bilateral involvement, n (%)	18 (69.2)	9 (64.3)	9 (75.0)
Systemic symptoms, n (%)			
Recurrent oral ulcers	26 (100.0)	14 (100.0)	12 (100.0)
Genital ulcers	10 (38.5)	6 (42.9)	4 (33.3)
Skin lesions	17 (65.4)	10 (71.4)	7 (58.3)
Erythema nodosum	11 (42.3)	9 (64.3)	2 (16.7)
Pseudofolliculitis	7 (26.9)	2 (14.3)	5 (41.7)
Arthritis	3 (11.5)	2 (14.3)	1 (8.3)
Perianal abscess	1 (3.8)	0	1 (8.3)
Thrombophlebitis	1 (3.8)	0	1 (8.3)
Uveitis type (44 eyes), n (%)			
Posterior uveitis	18 (40.9)	9 (39.1)	9 (42.9)
Panuveitis	26 (59.1)	14 (60.9)	12 (57.1)
Number of immunosupressants, M (IQR)	1 (1–2)	1 (1–2)	2 (1–3)
Concomitant immunosupressants, n (%)			
Cyclosporine-A	19 (73.1)	10 (71.4)	9 (75.0)
Azathioprine	10 (38.5)	7 (50.0)	3 (25.0)
Cyclophosphamide	5 (19.2)	1 (7.1)	4 (33.3)
Thalidomide	3 (11.5)	0	3 (25.0)
Mycophenolate mofetil	2 (7.7)	0	2 (16.7)
Tacrolimus	1 (3.8)	1 (3.8)	0
Methotrexate	1 (3.8)	0	1 (8.3)

BD, Behçet’s disease; BU, Behçet’s uveitis; IFN-α2a, interferon alpha-2a; CsA, cyclosporine-A.

Prior to enrollment, all patients were treated with corticosteroids in combination with a median of 1 immunosuppressant (range, 1–3). The median dose of prednisolone was 20.0 mg/day (range, 15.0–40.0 mg/day). The baseline immunosuppressive agents taken by patients included CsA (19 patients, 73.1%, median dose 125 mg/day), AZA (10 patients, 38.5%, median dose 100 mg/day), CTX (5 patients, 19.2%, median dose 100 mg/day), THD (3 patients, 11.5%, median dose 2 mg/day), MMF (2 patients, 7.7%, median dose 125 mg/day), TAC (1 patient, 3.8%, dose 2 mg/day), and MTX (1 patient, 3.8%, dose 15 mg/week). The IFN-α2a and CsA groups were not significantly different in basic demographic data, baseline clinical features, and treatments.

### Treatment Response

Of the 26 patients, 20 (76.9%) responded (complete and partial remission) to IFN or CsA treatment (Table 2). Specifically, 12/14 (85.7%) patients responded to IFN-α2a treatment, while 8/12 patients (66.7%) responded to CsA treatment (*p* = 0.365). Notably, complete remission (no relapse within the 12-month follow-up period) was achieved in 7 (50.0%) patients in the IFN-α2a group, compared to only 3 (25.0%) patients in the CsA group (*p* = 0.248). Of those patients who completely responded to the therapy, the duration between the therapy initiation to a complete absence of ocular inflammation was 3.3 and 7.0 months in IFN-α2a and CsA group, respectively (*p* = 0.023). On the other hand, for incomplete responders and nonresponders who suffered further uveitis attacks during the study period, the relapses occurred on average 4.7 ± 3.7 and 4.8 ± 2.2 months after IFN-α2a and CsA initiation, respectively (*p* = 0.966).

**TABLE 2:** Efficacy outcomes of the 26 refractory BU patients treated with IFN-α2a and CsA.

	Total (n = 26)	IFN-α2a (n = 14)	CsA (n = 12)	*p*
Treatment response, n (%)				
Complete remission	10 (38.5)	7 (50.0)	3 (25.0)	0.248
Partial remission	10 (38.5)	5 (35.7)	5 (41.7)	1.000
Treatment failure	6 (23.1)	2 (14.3)	4 (33.3)	0.365
Time to achieve complete remission (months) (10 eyes), $\bar{x}$± s	4.4 ± 2.5	3.3 ± 1.4	7.0 ± 3.0	**0.023**
Duration of relapse-free (months) (16 eyes), $\bar{x}$± s	4.8 ± 2.8	4.7 ± 3.7	4.8 ± 2.2	0.966
Baseline prednisone dose (mg/day), M (IQR)	20.0 (19.4–30.0)	20.0 (16.9–24.4)	20.0 (20.0–30.0)	0.207
Endpoint prednisone dose (mg/day), M (IQR)	15.0 (15.0–30.0)	15.0 (15.0–32.5)	25.0 (15.0–37.5)	0.432
Baseline LogMAR BCVA (44 eyes), M (IQR)	0.96 (0.17–1.40)	0.52 (0.10–1.00)	1.00 (0.61–1.52)	0.147
Endpoint LogMAR BCVA (44 eyes), M (IQR)	0.56 (0.00–1.20)	0.22 (0.00–0.92)	0.92 (0.31–1.70)	**0.031**
Distribution of low BCVA in baseline (44 eyes), n (%)				
20/50 or worse	33 (75.0)	16 (69.6)	17 (81.0)	0.494
20/200 or worse	22 (50.0)	9 (39.1)	13 (61.9)	0.227
LogMAR BCVA change rate (44 eyes), n (%)				
Improved ≥0.2LogMAR	12 (25.0)	8 (34.8)	4 (19.0)	0.318
Improved <0.2LogMAR	10 (20.5)	6 (26.1)	4 (19.0)	0.724
Stability	9 (25.0)	3 (13.0)	6 (28.6)	0.272
Deteriorated	13 (29.5)	6 (26.1)	7 (33.3)	0.744
Baseline BOS24 score (44 eyes), M (IQR)	5 (3–7)	5 (3–7)	5 (3.5–6.5)	0.803
Endpoint BOS24 score (44 eyes), M (IQR)	1 (0–4.75)	1 (0–3)	2 (0–6)	0.124
BOS24 score change rate, n (%)				
Improved	32 (72.7)	20 (87.0)	12 (57.1)	**0.042**
Stability	5 (11.4)	1 (4.3)	4 (19.0)	0.176
Deteriorated	7 (15.9)	2 (8.7)	5 (23.8)	0.232

LogMAR, logarithm of the minimum angle of resolution; BCVA, best-corrected visual acuity; BOS24: Behçet’s disease ocular attack score 24; IFN-α2a: interferon alpha-2a; CsA: cyclosporine-A. Bold values: p < 0.05.

### Effect on Visual Acuity

The analysis included 23 eyes in the IFN-α2a group and 21 eyes in the CsA group with refractory BU.

The baseline LogMAR BCVA was 0.52 (0.10–1.00) in the IFN-α2a group and 1.00 (0.61–1.52) in the CsA group (*p* = 0.147). BCVA equal or below 20/50 and 20/200 were found in 16 eyes (69.6%) and 9 eyes (39.1%) in the IFN-α2a group, compared to 17 eyes (81.0%) and 13 eyes (61.9%) in the CsA group (*p* > 0.05), respectively.

**FIGURE 2:**
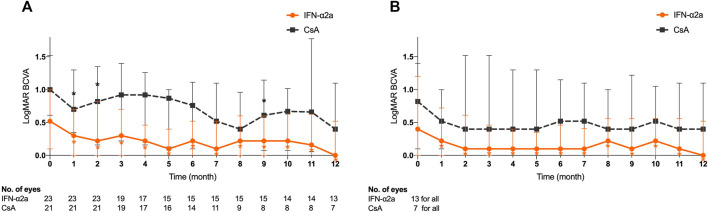
Changes in logarithm of the minimum angle of resolution (LogMAR) best-corrected visual acuity (BCVA) for eyes treated with interferon alpha-2a (IFN-α2a) and cyclosporine-A (CsA). **(A)** Changes in median LogMAR BCVA for all eyes treated with IFN-α2a (n = 23) and CsA (n = 21) during the 12-month follow-up. **(B)** Changes in LogMAR BCVA for eyes with complete remission treated with IFN-α2a (n = 13) and CsA (n = 7) during the 12-month follow-up. Data are shown as the median and IQR. *: *p* < 0.05.

Of the 23 enrolled eyes in the IFN-α2a group, the improvement in LogMAR BCVA started at the first month’s visit after treatment initiation (*p* < 0.001), and this beneficial effect sustained to the endpoint visit (*p* = 0.026) (Figure 2A). In contrast, compared with the baseline level, LogMAR BCVA of 21 eyes in the CsA group did not show either continuous improvement during the follow-up period or at the endpoint visit (*p* > 0.05). Notably, at the end of the study, the median LogMAR BCVA increased to 0.22 (0.00–0.92) and 0.92 (0.31–1.70) in the IFN-α2a group and CsA group, respectively (*p* = 0.031).

Furthermore, of all eyes in the IFN-α2a group, BCVA improved ≥0.2 LogMAR from study initiation in 8 eyes (34.8%), improved but less than 0.2 LogMAR in 6 eyes (26.1%), remained stable in 3 eyes (13.0%), and worsened in 6 eyes (26.1%). On the other hand, in the CsA group, BCVA improved by ≥0.2 logMAR in only 4 eyes (19.0%), but it stabilized and deteriorated in 6 eyes (28.6%) and 7 eyes (33.3%), respectively.

Among 20 eyes that responded (complete and partial remission) to the IFN-α2a therapy, LogMAR BCVA was 0.60 (0.17–1.30) at baseline, and significantly increased 1 month after treatment initiation (*p* = 0.001), remained statistically significant at every follow-up visit, and eventually improved to 0.31 (0.00–0.98) at study endpoint (*p* = 0.020). Meanwhile, in the complete remission subgroup, a total of 13 eyes showed similar VA progression (Figure 2B). However, no such improvement was observed in either 7 complete remission or 7 partial remission eyes in the CsA group (*p* > 0.05).

### BOS24 Score in Patients With BU

**FIGURE 3:**
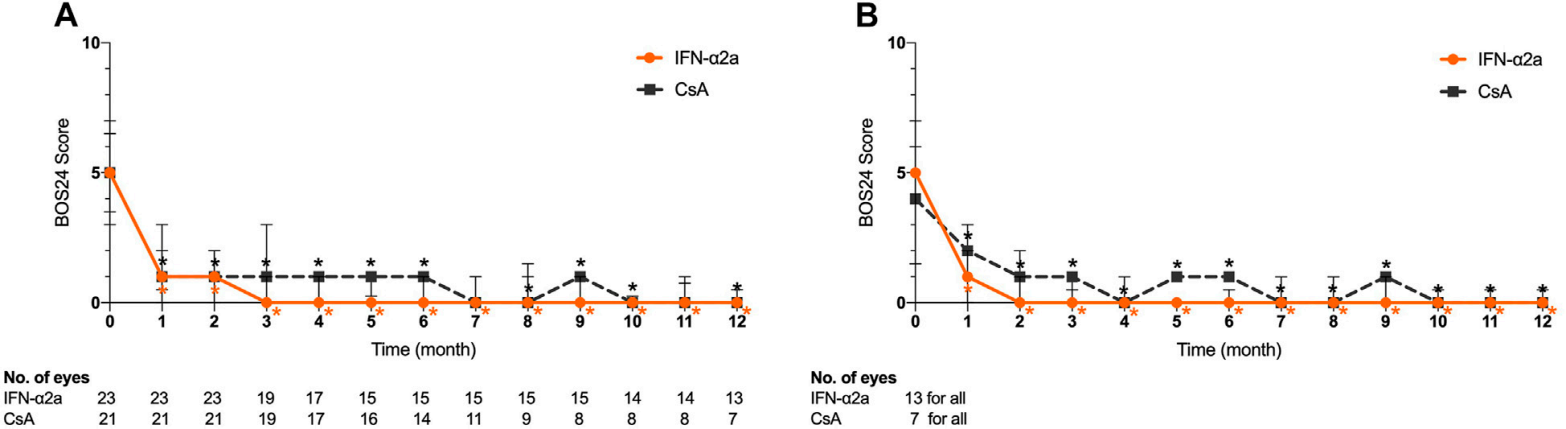
Changes in Behçet’s disease ocular attack score 24 (BOS24) for eyes treated with interferon alpha-2a (IFN-α2a) and cyclosporine-A (CsA). **(A)** Changes in median BOS24 score for all eyes treated with IFN-α2a (n = 23) and CsA (n = 21) during the 12-month follow-up. **(B)** Changes in BOS24 score for eyes with complete remission treated with IFN-α2a (n = 13) and CsA (n = 7) during the 12-month follow-up. Data are shown as the median and IQR. *: *p* < 0.05.

The median baseline BOS24 scores were 5 (3–7) and 5 (3.5–6.5) in the IFN-α2a and CsA groups, respectively (*p* = 0.803). Of all eyes in the IFN-α2a group, the BOS24 score showed a significant decrease 1 month after treatment initiation (*p* < 0.001) and remained low during the entire study period (*p* = 0.001) (Figure 3A). However, in the CsA group, statistically significant reductions in BOS24 scores were not observed in a few follow-up visits and the endpoint visit, as compared to the baseline. At the end of this study, the BOS24 score fell to 1 (0–3) and 2 (0–6) in the IFN-α2a and CsA groups (*p* = 0.124), respectively.

Moreover, at the endpoint visit, a decreased BOS24 score was obtained in 20 out of 23 eyes (87.0%) and 12 out of 21 eyes (57.1%) in the IFN-α2a and CsA groups, respectively (*p* = 0.042). In contrast, only 2 eyes (8.7%) showed a higher BOS24 score when relapse occurred during the IFN-α2a treatment period, while 5 eyes (23.8%) in the CsA group had an elevated score at the endpoint, indicating a more severe ocular inflammation status (*p* = 0.232).

Among eyes with complete and partial remission, the BOS24 score decreased over time in both the IFN-α2a and CsA groups (Figure 3B). Compared with the baseline BOS24 score, a significant BOS24 reduction was observed at monthly follow-up visits and was preserved at the final visit of the study (*p* < 0.05).

### Corticosteroid-Sparing Effect

After IFN-α2a or CsA treatment, the prednisolone dose was reduced in 8 (57.1%) and 5 (41.7%) patients at the end of the study, respectively (*p* = 0.695). The median corticosteroid dosage before enrollment was 20.0 mg (16.9–24.4) and 20.0 mg (20.0–30.0) per day in the IFN-α2a and CsA groups, respectively (*p* = 0.207). At the endpoint, the average dosage of corticosteroid was significantly decreased to 15.0 mg per day in complete remission patients treated with IFN-α2a (*p* = 0.024). Nevertheless, no obvious corticosteroid-sparing effects were observed in patients in the other subgroups, including partial remission and treatment failure patients in the IFN-α2a group, and all CsA subgroups (*p* > 0.05).

### Safety

The tolerance rate of both the IFN-α2a and CsA groups was 100% in this study. No treatment discontinuation was required because of the side effects. No serious adverse drug effects were observed. The incidence of adverse events in patients treated with IFN-α2a and CsA was 78.6% (11/14) and 66.7% (8/12), respectively (*p* = 0.665). Compliance with IFN-α2a was satisfactory. IFN-associated side effects, which were mild and reversible, included flu-like syndrome associated with fever, myalgia, and headache (at the initiation phase of the treatment) (71.4%; n = 10), mild elevation of serum liver enzymes (ALT and/or AST, 28.6%; n = 4), hair loss (28.6%; n = 4), skin disorders (erythema at injection site, reddish rash; 28.6%; n = 4), minor leukopenia (14.3%; n = 2), dryness of mouth (14.3%; n = 2), and mild depression (14.3%; n = 2). The side effects related to the CsA treatment were as follows: increased ALT/AST (33.3%; n = 4), increased uric acid (25.0%; n = 3), hyperlipidemia (25.0%; n = 3), hypertension (16.7%; n = 2), hematuria (16.7%; n = 2), and increased bilirubin (16.7%; n = 2). Hirsutism was observed in one female patient in the CsA group.

## Discussion

CsA has been one of the best-validated immunosuppressants for refractory eye disease in patients with BD (Chighizola et al., 2017). However, the beneficial effect of CsA was not sustained in the long term, with a high rate of side effects (BenEzra et al., 1988). On the other hand, accumulating evidence indicates that IFN-α is noticeably effective for refractory BU patients with a high tolerance rate (Kötter et al., 2003; Gueudry et al., 2008). Therefore, in the most recent EULAR recommendations (Hatemi et al., 2018), IFN-α is one of the recommended agents for patients with recurrent episodes of acute sight-threatening uveitis based on its efficacy in inducing rapid ocular inflammation remission, preventing recurrences, and maintaining useful vision in medium to long terms. To the best of our knowledge, this is one of the first clinical trials to address head-to-head comparisons between IFN-α and CsA. Another advantage of this study was the application of the BOS24 scoring system for disease activity of BU (Kaburaki et al., 2014), which has a low level of variability among different examined ophthalmologists and has been successfully applied in previous studies (Kaburaki et al., 2014; Tanaka et al., 2016).

In the literature, the dosage regimens of IFN and CsA vary among different clinical centers and study protocols. IFNα-2a is usually subcutaneously injected at doses ranging from 3 to 9 MIU, 3 to 7 times a week (Kötter et al., 2004), and CsA is orally administered at dosages ranging from 2 to 16 mg/kg/day (Whitcup et al., 1994; Evereklioglu, 2005). In our current study, the initial dose of IFNα-2a was 3 MIU daily for the first month, followed by 3 MIU every other day as the maintenance dose, based on experiences gained from our retrospective study (Shi et al., 2019). CsA was administered at a dosage of 200 mg/day (with an average of 2.7 m g/kg/day) during the entire study period, which was commonly prescribed for patients with refractory BU in our clinical practice.

A review of previous studies has revealed invariably high (78% to over 90%) response rates of IFN-α2a for treatment of BU (Krause et al., 2008; Hazirolan et al., 2013). The rate of patients who achieved complete remission, however, was quite different among investigations, ranging from 36.4% to 85.0% (Tugal-Tutkun et al., 2006; De Simone et al., 2020). The reported response rates of CsA are generally lower, ranging from 50% to 85% (Masuda et al., 1989; Murphy et al., 2005). In accordance with the literature, in our current 12-month study, the IFN-α2a group showed both higher response rates and complete remission rates than the CsA group (85.7% vs. 66.7% and 50% vs. 25.0%, respectively), indicating the superiority of IFN-α2a over CsA for long-term control of refractory BU.

The advantage of IFN-α2a over CsA was also reflected by the time to reach complete remission, and the improvements in visual function and disease severity, as indicated by LogMAR BCVA and BOS24 score, respectively. Our current study showed that the use of IFN-α2a treatment led to a significantly earlier complete remission in refractory BU patients than CsA treatment. Additionally, during the entire 12-month period, treatment with IFN-α2a can effectively achieve sustained disease control by markedly increasing visual acuity and reducing BOS24 score, regardless of whether the patients achieved complete remission. Consequently, at the endpoint of the study, more patients in the IFN-α2a group achieved a prominent visual acuity improvement with amelioration of intraocular inflammation, as compared to the CsA group. Therefore, this randomized prospective comparative clinical trial provides multiple lines of evidence suggesting that in the treatment of refractory BU, IFN-α2a treatment can not only reduce the dosage of glucocorticoids but also display superiority in inducing rapid disease remission and maintaining disease quiescence in 12 months.

Our study also revealed generally favorable safety profiles for both IFN-α2a and CsA regimens. Although adverse effects were recorded in 78.6 and 66.7% of the patients in the IFN-α2a and CsA groups, respectively, they were all reversible and well tolerated. The most frequent side effects of IFN-α2a and CsA were flu-like symptoms (71.4%) and renal toxicity (33.3%), respectively, which are in accordance with previous studies (Chighizola et al., 2017; Shi et al., 2019). We also calculated the 1-year costs of our IFN-α2a and CsA regimens, which were approximately $1,050 and $1,600, respectively. Therefore, IFN-α2a treatment is more cost-effective than CsA treatment for patients with refractory BU in China.

This study has some limitations. First and most importantly, the sample size was relatively small and inadequate for more detailed analyses and comparisons. The approval of adalimumab for refractory non-infectious uveitis in March 2020 in China and the COVID-19 pandemic have made it difficult to recruit participants further. Second, we noticed that there was a difference in the baseline BCVA between the IFN-α2a and CsA groups, although this disparity was not statistically significant. Third, the current study period was not long enough to evaluate the long-term efficacy of IFN-α2a and CsA treatment. It would be of higher clinical qualifications to conduct the study over a longer time span.

In conclusion, this randomized, controlled, prospective clinical trial provides multiple lines of evidence suggesting that IFN-α2a is superior to CsA when combined with glucocorticoid for refractory BU during a study period of 12 months. Compared to CsA, IFN-α2a induces a higher rate of treatment response, a significantly better improvement in visual acuity, and a more stable disease remission in 12 months for refractory BU.

## Data Availability

The raw data supporting the conclusions of this article will be made available by the authors, without undue reservation.
